# Supplementary material for: Post-whaling shift in mating tactics in male humpback whales
Source: Commun Biol. 2023 Feb 16;6:162. doi: 10.1038/s42003-023-04509-7 (PMC9935900; doi:10.1038/s42003-023-04509-7)
Supplement: Supplementary file 2 — Supplementary Information [file 42003_2023_4509_MOESM2_ESM.pdf]

Estimating the proportion of migrating humpbacks that are male

Rebecca Dunlop[1]

[1]School of Biological Sciences, University of Queensland, St Lucia, Brisbane, Australia, QLD 4072.

The main study relies on the estimation of the proportion of migrating adults that were assumed to be male given it was not logistically possible to sample all migrating animals to ascertain their sex. The following presents two analyses. The first is the results of a biopsy study carried out in this field site in 2014 and 2015. The second is a sensitivity analysis of the assumptions made according to group composition and the effect this has on male proportion.

Supplementary Methods

Sexing using biopsy samples

Behavioural data collection

Adult humpback whales were sampled during the 2014/2015 field effort. Groups of whales were approached by the research vessel for the purposes of biopsy sampling. Before sampling took place, behavioural observations were undertaken for at least 20 minutes. During this time, all animals within the group were allocated an individual identification name based on the shape and coloration of their dorsal fin. Photographs were taken of each adult's dorsal fin, fluke (if presented) and any other obvious markings. Using these individual identification markings, during a surfacing event, data such as the position of each animal in the group, and its general behaviour, was recorded (see Table 1). Animals usually kept the same position within the group, and displayed the same suite of behaviours, throughout the observation period, meaning these data could be summarised for all individuals within the group. During this observation period, the group composition was also described, as well as changes to this composition due to joining of new whales.

Following this observation period, animals were biopsied (see later for description). Photographs and a video recording were also taken to identify the whale being biopsied. This enabled later matching if there was any confusion as to which animal within the group was being sampled. Because biopsies involve making close (within 30 m) approaches to the group, it becomes increasing difficult to sample group members as time goes on. Therefore, only on rare occasions were all group members of larger groups sampled.

Six different group compositions were encountered: lone single animal, female-calf pair, two adult group, three adult group, two adults and one calf, up to six adults and one calf. During the pre-biopsy observation period, based on each adult's behaviour within the group (Supplementary Table 1), each group member was allocated a sex (likely male or likely female). Lone non-singing adults, and adults within groups where there was no obvious mate guarding behaviours, were not allocated a sex as it was not possible to tell from their behaviour.

Supplementary Table 1. Description of individual behaviours within a group and the allocated sex based on these observed behaviours.

**Group composition**	**Assumed sex based on behaviour**
Lone adult	Male if singing, unknown if not singing
Female-calf	Adult assumed to be female
Female-calf-escort	Adult surfacing with the calf assumed to be female with the other adult assumed to be a male escort
Adult pair	Sex not assigned in field
Three adult group	Assumed these comprised of two leading adults (that surface together) and one smaller trailing adult assumed to the secondary escort (male). Of the two leading adults, the one preforming mate guarding behaviours (chasing and blocking the secondary escort) was assumed to be the primary escort (male), and the other was assumed to be female. If there was no clear mate guarding behaviours, and/or there was no obvious lead animal(s), adults were not allocated a sex.
Joining animal	Assumed to be male
Female-calf- and 2 or more escorts	Adult surfacing with the calf assumed to be female, all other adults assumed to be the male escorts with the primary male escort surfacing with the female and leading the group, and the secondary escorts following. Secondary escorts were usually smaller than the primary escort.

More than three adults	As with the three-adult group. Lead pair assumed to be the primary male escort and female. Trailing animals assumed to secondary male escorts. The primary escort usually performed mate-guarding behaviours and surfaced with the assumed female.

Biopsy sample collection

Biopsy sampling was conducted after the observation period using a PAXARMs remote biopsy system (PAXARMs New Zealand Ltd). Collected tissue samples were rinsed with distilled water, stored in 5 mL polystyrene tubes (LBSSP2002, ThermoFisher, Australia), placed briefly on ice (1 – 8 hours) and then transferred to -20°C at the end of a day. The first sampled animal was not a targeted group member, but the first animal to come close enough to the vessel to enable sample collection. This animal was then identified by the onboard observers from its dorsal fin and, if available, fluke as described above. From then on, animals within the group were purposely targeted based on their individual identification markings. This was to ensure the same animal was not re-sampled and as many animals as possible within the group were sampled. If there was confusion as to which group member was sampled, sampling videos were matched to group members based on their identification markings post-sampling. Once the group displayed obvious avoidance behaviours, sampling ceased. All tissue samples were then transferred into -80°C at the end of a field season (3 – 6 weeks duration).

Genetic tests were conducted on all skin samples to determine the sex of each whale (Mingramm et al. 2020). These analyses were performed by the Animal Genetics Lab (UQ) and the Australian Antarctic Division.

Land-based data collection

Land-based observations of all migrating groups passing through the study area were collected daily (continuously 7am to 5pm, weather permitting) from an elevated survey point, Emu Mountain (73m). A theodolite (Leica TM 1100) was used in conjunction with a notebook computer running either *CYCLOPES* or *VADAR* software (E. Kniest, Univ. Newcastle, Australia) to track the migrating groups visually in real-time and record their behaviour. From 2003 onwards, groups were observed *ad lib* and tracked by teams of five

people; one person on the theodolite, one on data entry on a laptop connected to the theodolite, and three scanning observers with compass-reticule binoculars (7x50; Kingslux, Fujinon Mariner, Bushnell). When whale groups surfaced, the observers called the behaviour, compass bearing and angle from the group to the horizon (in reticules), and the theodolite operator attempted to measure the group's position at least every 10 min. Thus, each observation included a group identification letter, the time, group behaviour, group size, whether a calf was present, direction of travel, and group location, either using reticular measurement or more accurate theodolite measurement. Travelling groups typically surfaced every 3 – 5 mins. Not all surfacings were observed but, for most, enough theodolite locations were made to allow the development of a reliable track, particularly as the whales were mostly heading in a relatively constant direction. Weather was noted hourly.

Land-based data consolidation

Group data from each survey day were summarised (n = 137 days). The summary included the total number of observed adult whales, total number of observed calfs, and the total number of each group composition (lone animal, adult pair, female-calf, female-calf-escort, three adults, female-calf-two/three/four escorts and four/five/six adult group. As above, it was assumed all female-calf pairs were comprised of an adult female and her calf (16% of groups observed), and female-calf-escort groups (8% of groups observed) were comprised of an adult female, a calf, and a male escort. For groups comprising of three or more adults and groups comprising of female-calf-multiple escort groups, it was assumed they contained one adult female with the rest of the adults being male escorts. Since this latter group category comprised of 8% of the groups, changing this sex allocation would have had little effect on the final proportion of animals assumed to be male. Therefore, the remainder of the analysis assess the effect of sex allocation of lone animals (46% of groups) and adult pairs (22% of groups) on the estimated proportion of migrating adults assumed to be male.

Supplementary Results

Sexing using biopsy samples

A total of 101 samples were collected from 66 different groups. There were also three sampled joining animals to these group; one animal joined a lone singing whale, one joined a three-adult group, and one joined a four-adult group. Overall, 30 females and 71 males were sampled equating to a male dominated sex ratio of 1: 2.2, similar to Brown and Corkeron's (1995) ratio of 1: 2.4. However, Brown and Corkeron (1995) noted a potential bias in sampling in that large (male-dominated) competitive groups were more visible and therefore more likely to be sampled. The same bias applies in this study, as well as the fact that female-calf pairs were not sampled as often given the adult was known to be female. Therefore, the sex ratio, though likely to be male dominated, is potentially lower than 1: 2.2.

Of the 101 samples collected, 91 (90%) were assigned a sex in the field based on their behaviour. Unassigned animals included lone non-singing animals (3), samples from adult pairs (5), and two samples from a three-adult group where there was no obvious female, primary, and secondary escort roles based on their behaviour (Supplementary Table 2). Of the 91 assigned a sex, only one was incorrectly assigned meaning 89% of the samples were correctly sexed based on the animal's behaviour and the group composition. The incorrect sample was from a female-calf-escort group, where the female (mother) was incorrectly allocated the escort role. For this group, the other animal had been sampled and allocated 'female', suggesting the female was mistakenly sampled twice.

All group members were sampled in eight groups; six female-calf-escort groups, a female-calf-two escort group, and a female-calf- four escort group. All six female-calf-escort groups were comprised (as expected) of a female and male adult. All escorts within the two- and four-escort groups were (as expected) male with one female.

Supplementary Table 2. The number of sampled groups and animals categorised by group composition. Of these, the number of animals that were assigned male of female in the field based on their behaviour, and of these, the number that were correctly assigned based on the biopsy result. The last column represents the number of individuals that were not assigned a sex in field.

**Group composition**	**no. groups**	**no. animals**	**male**		**female**		**unassigned**
			**assigned**	**result**	**assigned**	**result**	
Lone adults	7	7	4	7	0	0	4
Joining adults		3	3	3	0	0	0
Female-calf	12	12	0	0	12	12	0
Female-calf-escort	22	28	17	18	10	10	0
Adult pairs	5	5	0	4	0	1	5
Three adults	10	17	11	13	4	4	2
Female-calf- and 2 or more escorts	6	15	12	12	3	3	0
More than three adults	4	14	14	14	0	0	0

Estimating the proportion of adults that were assumed to be male

The following formula was used to estimate the proportion of migrating adults assumed to be male for each survey day:

Male proportion = (lone adults * a) + ((adult pairs * 2)* b) + escorts / total number of adults

where a and b were different proportions of lone adults and adult pairs assumed to be male. In this analysis, a ranged from 0.3 (30% male) to 0.9 (90% male) and b ranged from 0.5 (all pairs were comprised of a female and a male) to 0.8 (80% of animals within pairs were comprised of two males) given male adults, on relatively rare occasions, have been observed to join singing whales to form an adult pair in this study site (Smith et al. 2008). Changing the a and b variables as described above gave 28 iterations for the sensitivity analysis.

A total of 137 days used for this analysis: 28 from 1997, 75 from 2003 and 2004, 14 from 2008, 20 from 2014 and 2015. Overall, the estimated proportion of migrating males per day (running all 28 iterations for each of the 137 days) ranged from 0.12 to 0.9.

The results for the 137 days were then averaged to give an estimated proportion of migrating males assumed to be male from each of the 28 iterations. Average male proportions ranged from 0.4 (30% of lone animals assumed to be male and 50% of adults in adult pairs assumed to be male), to 0.69 (90% of lone animals assumed to be male and 80% of adults in adult pairs assumed to be male).

Given other studies, including this biopsy study, that found a male bias in the proportion of migrating adults found to be male, all iterations in which the proportion of migrating adults assumed to be male came to less than 0.5 were eliminated. This reduced number of iterations to 12, with variable *a* ranging from 0.5 to 0.9 and variable *b* ranging from 0.6 to 0.8. For these results, the proportion of migrating adults assumed to be male ranged from 0.54 to 0.69.

To carry out the sensitivity analysis, one of the variables was held constant while the other varied. First, variable *b* (proportion of males within adult pairs) was held constant at 0.7, and the analysis re-run with variable *a* (proportion of lone animals assumed to be male) ranging from 0.5 to 0.9. This gave an average proportion of migrating adults assumed to be male ranging from 0.53 to 0.66 (Supplementary Figure 1), i.e., a variation in male proportion of 13%. It is unlikely that 90% of lone adults are male given this would mean most females were with a calf and/or being escorted during the survey period with very few females being alone. It is also unlikely that 50% of the lone adults are male given the prevalence of breeding behaviours such as adults joining other groups and singing whales. The most reasonable proportion of lone adults likely to be male was therefore between 0.6 and 0.8. Variable *a* was set at 0.7 in the subsequent analysis.

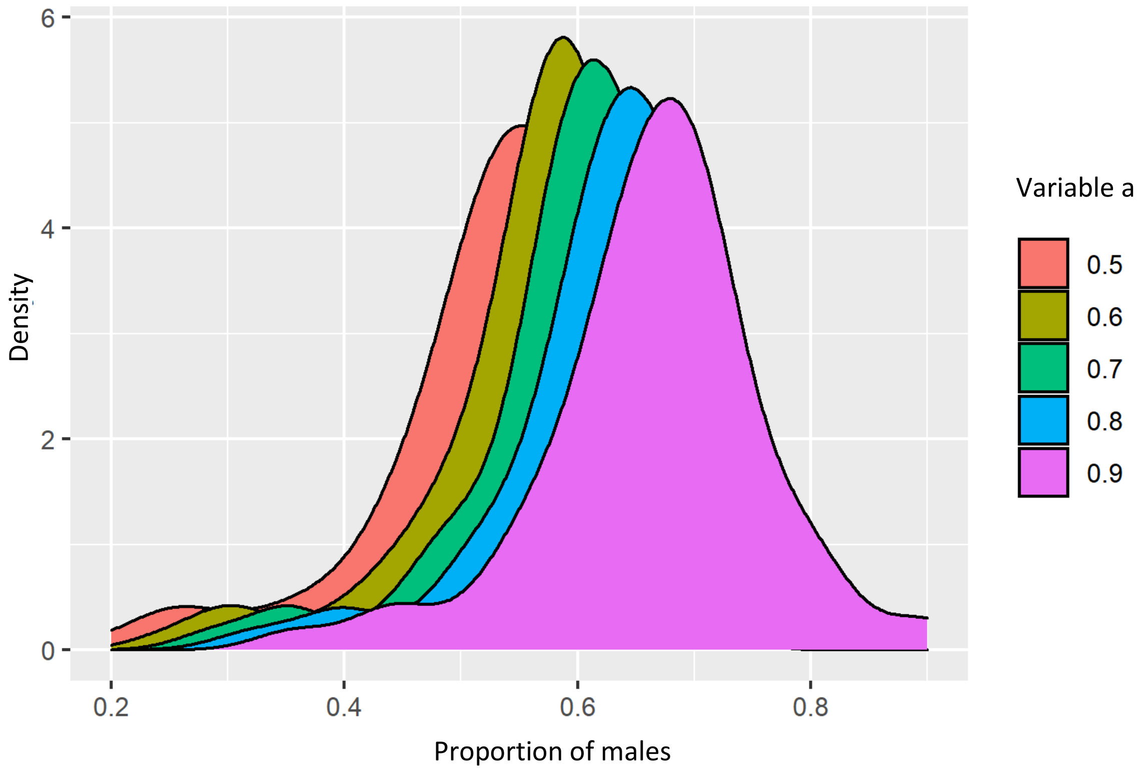

Supplementary Figure 1. The distribution of estimated male proportions where the proportion of males within adult pairs was assumed to be 0.7 and the proportion of lone adults assumed to be male varied between 0.5 and 0.9.

In terms of assumptions for adult pairs, studies have also found a mix of male-male and male-female adult pairs (with rare occurrence of female-female pairs). If assuming adult pairs were also male biased (i.e., setting variable *b* to range between 0.6 and 0.8), and holding variable *a* constant (at 0.7), the average proportion of migrating animals assumed to be male ranged from 0.57 to 0.63 (Supplementary Figure 2). Because adult pairs accounted for 22% of the groups observed, per day, this changed the number of adults assumed to be male per day by one.

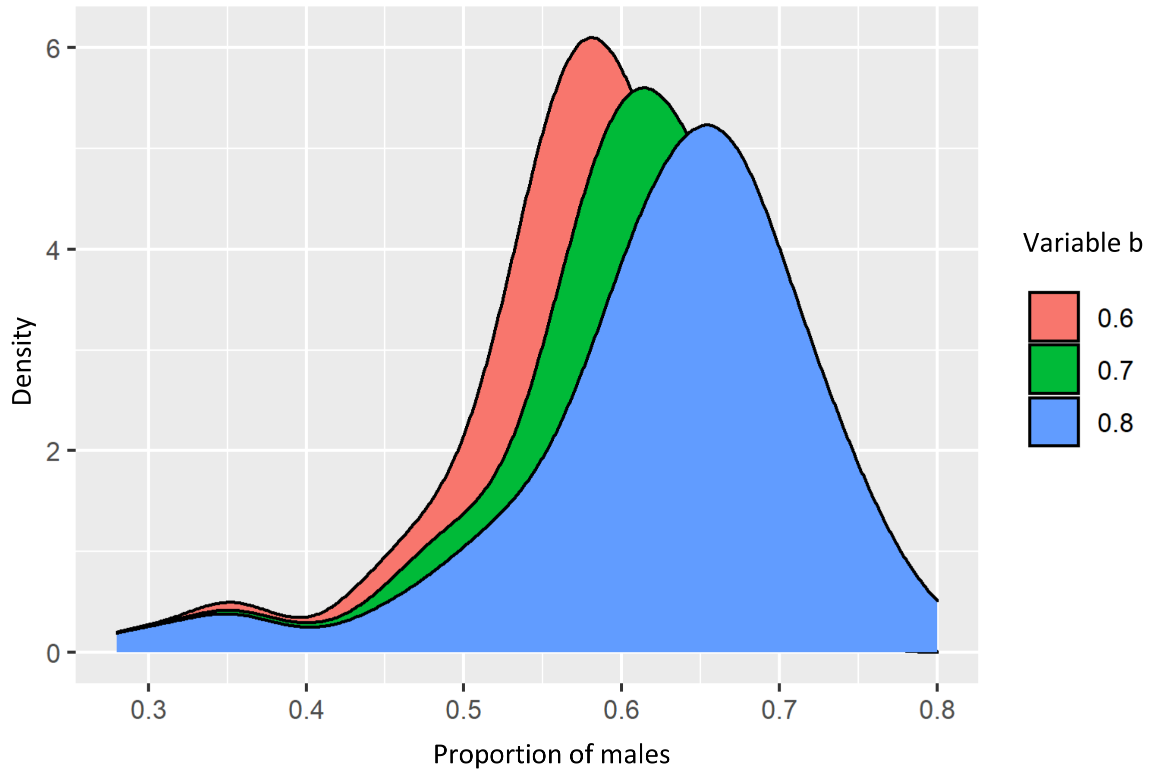

Supplementary Figure 2. The distribution of estimated male proportions where the proportion of males within lone animals was assumed to be 0.7 and the proportion adults within pairs assumed to be male varied between 0.6 and 0.8.

Supplementary Note

The following outlines the assumption of sex based on group composition made within the main study and details how these biopsy results generated these assumptions.

1. All joining non-singing animals were assumed to be male

Results from this study suggest this is a valid assumption. Female-calf pairs sometimes join with each other temporarily, but there is no evidence to suggest single females join groups.

2. Female-calf pairs were being escorted by males

A total of 22 female-calf-escort groups, and six female-calf- 2+ escorts groups were sampled in this study. Of these, eight had all group members sampled. Fully sampled groups met prior expectations of sex, in that there was never more than one female in the group, and all escorts were male. In groups that were not fully sampled, all contained group members with correctly assigned male escorts and the female based on individual behaviour within the group.

3. Groups of multiple adults were assumed to be comprised of a single female, principal male escort and secondary male escorts or challengers

A total of 10 three- adult groups, two four- adult, one five- adult and a six- adult group were sampled. None of these groups were completely sampled, but all groups contained group members with correctly assigned male escorts and a female being escorted. No sampled group containing more than one female.

4. Lone animals not involved in any group interactions, and not singing, were given a 70% chance of being male.

Of note is that all sampled lone animals were male. However, during this study, male singing whales were targeted (accounting for 4 of the 7 sampled lone whales). This was the most common group type, and the one with the greatest unknown in terms of sex allocation. See sensitivity analysis below on effect of variation in sex allocation of these lone animals on the final estimation of the proportion of migrating adults that were assumed to be male.

5. Adult pairs

This was the second most common group type, but unfortunately no complete adult pair groups were biopsied. These are likely to be a mix of male-female pairs and male-male dyads (Brown and Corkeron 1995, Smith et al. 2008). See sensitivity analysis below on effect of variation in sex allocation of these adult pairs on the final estimation of the proportion of migrating adults that were assumed to be male.

For the second analysis, the proportion of migrating adults assumed to be male, the most sensitive variable was the allocated proportion of lone animals assumed to be male. The most likely range of values, based on behavioural observations and previous sexing studies (Brown and Corkeron 1995; Smith et al. 2008), is between 0.6 and 0.8 (with an estimated 7% variation in male proportion). This translated to, on average, a difference of two adults allocated male per day depending on which proportion was used. For this study, 0.7 was used. Setting both the lone adult and adult pair proportion at 0.7 gave a final average of proportion of migrating adults assumed to be male of 0.60 with a sex ratio of 1: 1.5.

To check for any systematic bias, we assumed the proportion of migrating animals that were male should remain stable over time. The above formula was then used to estimate the proportion of migrating animals assumed to be male for day and compared between study years using a general linear model (with the proportion of estimated males as the response

variable and year as the fixed effect and assuming a quasipoisson distribution). There was no evidence of a significant change in the proportion of migrating animals allocated male over time (t = -1.323, P = 0.188; t = -0.036, P = 0.971 and t = -1.244, P = 0.216 for 2003/2004, 2008, and 2014/2015 compared to 1997).

When assessing the number of males within the social circle of focal males, most animals could be allocated a sex based on the group composition and their behaviour (e.g., female with a calf, female-calf being escorted, lone male joining a group containing a female, singing whale, adult (likely) male pair joining a group containing a female). Lone adults of unknown sex, and adults within adult pairs, were given a 70% chance of being male. There was never an occasion where all adults within a focal male's social circle were of unknown sex and adult pairs (of unknown sex) were rare.

References

Brown, M. & Corkeron, P. Pod characteristics of migrating humpback whales (*Megaptera novaeangliae*) off the east Australian coast. *Behaviour* **132**, 3-4, 163-170 (1995).

Mingramm, F. M. J., Keeley, T., Whitworth, D. J. & Dunlop, R. A. The influence of physiological status on the reproductive behaviour of humpback whales (*Megaptera novaeangliae*). *Hormones and Behavior* **117** (2020). https://s10-doi-org.ezproxy.library.uq.edu.au/j.yhbeh.2019.104606 \

Smith, J. N., Goldizen, A. W., Dunlop, R. A. & Noad, M. J. Songs of male humpback whales, *Megaptera novaeangliae*, are involved in intersexual interactions. *Animal Behaviour* **76**, 467-477 (2008). https://s10-doi-org.ezproxy.library.uq.edu.au/j.anbehav.2008.02.013
