# Supplementary material for: Post-whaling shift in mating tactics in male humpback whales
Source: Commun Biol. 2023 Feb 16;6:162. doi: 10.1038/s42003-023-04509-7 (PMC9935900; doi:10.1038/s42003-023-04509-7)
Supplement: Supplementary file 3 — Reporting Summary [file 42003_2023_4509_MOESM3_ESM.pdf]

Corresponding author(s): COMMSBIO-22-2574-T

Last updated by author(s): 27/07/2022

Reporting Summary

Nature Research wishes to improve the reproducibility of the work that we publish. This form provides structure for consistency and transparency in reporting. For further information on Nature Research policies, see our Editorial Policies and the Editorial Policy Checklist.

Statistics

For all statistical analyses, confirm that the following items are present in the figure legend, table legend, main text, or Methods section.

n/a	Confirmed	
☐	☒	The exact sample size (*n*) for each experimental group/condition, given as a discrete number and unit of measurement
☐	☒	A statement on whether measurements were taken from distinct samples or whether the same sample was measured repeatedly
☐	☒	The statistical test(s) used AND whether they are one- or two-sided<br>*Only common tests should be described solely by name; describe more complex techniques in the Methods section.*
☐	☒	A description of all covariates tested
☐	☒	A description of any assumptions or corrections, such as tests of normality and adjustment for multiple comparisons
☐	☒	A full description of the statistical parameters including central tendency (e.g. means) or other basic estimates (e.g. regression coefficient) AND variation (e.g. standard deviation) or associated estimates of uncertainty (e.g. confidence intervals)
☐	☒	For null hypothesis testing, the test statistic (e.g. *F*, *t*, *r*) with confidence intervals, effect sizes, degrees of freedom and *P* value noted<br>*Give P values as exact values whenever suitable.*
☒	☐	For Bayesian analysis, information on the choice of priors and Markov chain Monte Carlo settings
☒	☐	For hierarchical and complex designs, identification of the appropriate level for tests and full reporting of outcomes
☐	☒	Estimates of effect sizes (e.g. Cohen's *d*, Pearson's *r*), indicating how they were calculated

Our web collection on statistics for biologists contains articles on many of the points above.

Software and code

Policy information about availability of computer code

Data collection	Cyclopes software (developed by Eric Kniest, Univ. Newcastle, Australia, available on request),
Data analysis	No custom software used

For manuscripts utilizing custom algorithms or software that are central to the research but not yet described in published literature, software must be made available to editors and reviewers. We strongly encourage code deposition in a community repository (e.g. GitHub). See the Nature Research guidelines for submitting code & software for further information.

Data

Policy information about availability of data

All manuscripts must include a data availability statement. This statement should provide the following information, where applicable:

- Accession codes, unique identifiers, or web links for publicly available datasets
- A list of figures that have associated raw data
- A description of any restrictions on data availability

Raw data has been uploaded onto the University of Queens;and's Research Data Management System and is published in UQ eSpace. The link is available on request. There are no restrictions on data availability.

Field-specific reporting

Please select the one below that is the best fit for your research. If you are not sure, read the appropriate sections before making your selection.

☐ Life sciences ☐ Behavioural & social sciences ☒ Ecological, evolutionary & environmental sciences

For a reference copy of the document with all sections, see nature.com/documents/nr-reporting-summary-flat.pdf

Ecological, evolutionary & environmental sciences study design

All studies must disclose on these points even when the disclosure is negative.

Study description

Four datasets, equating to four post-whaling timeframes, were used for this study: 1997 (32 years post-whaling), 2003/2004 (38/39 years post-whaling), 2008 (43 years post-whaling and 2014/2015 (49/50 years post-whaling). For the analysis of individuals, 117 unaccompanied focal males were selected from the 2003/2004 dataset. When first observed, they were either singing (86) or not singing (31). For each unaccompanied focal male, the number of, and roles, of other presumed males within 5 km radius from the focal whale was used as a measure of local male density. The first analysis aimed to determine if the likelihood of first observing the focal individual as a singing or non-singing male was significantly related to local male density, as determined by the number of males within a 5 km radius, termed social circle. Singing whales were allocated a 0 and non-singing whales were allocated a 1. A generalised linear model structure was used, assuming a binomial distribution. Likely males within their social circle were divided into non-singing and singing males (to delineate tactics) and these were included as the two covariates. Of these, 40 males were selected for further analysis given they were observed to switch tactic. Their social circle was quantified when they were first observed along and singing and again as soon as they stopped singing.To test if focal males were more likely to switch tactic in increasing local male density (the number of non-singing and singing males in the area), a generalised mixed model structure was used, assuming a binomial distribution, and including focal male ID as the random effect to accounted for repeated measures within animals. For the population-level analysis, daily observations (N = 123), which comprised of 10 hours of combined land-based and acoustic observations were used. Here, the number of singing whales was counted using the acoustic recordings and concurrent sightings of the singing whales. The number of singers per day was then correlated against the number of males migrating through the area in a day within each timeframe (categorised as 1997, 2003/2004, 2008, 2014/2015). The response variable was the number of singers. As these were count data, ranging between 0 and 6 singers, with evidence of underdispersion, a quasi-Poisson distribution was assumed, where the p-values and confidence intervals were adjusted using an estimated dispersion parameter. A generalised additive model (gam) structure was used given it does not assume a fixed relationship between the response variable and covariates . The two covariates were timeframe, and the number of migrating males within each timeframe, where the number of migrating males was the smooth term modelled separately for each timeframe. Finally, a payoff score for an individual non-singing, and singing, male was created per day based on the number of observed non-singing joins (likely benefit) and additional joins (likely cost), number observed singing joins (likely benefit) and additional joins (likely cost), corrected for the number of migrating males to give an adjusted score per individual. To test if one tactic was more successful than the other within each timeframe, t a zero-inflated negative binomial distribution was assumed due to overdispersion and an excess of zeros where tactic was this predictor variable and the random effect of day was included to account for the repeated measures (i.e., the payoff score for each tactic was estimated for each day resulting in a paired comparison). Then, estimated daily payoff scores were then compared between timeframes assuming a zero-inflated negative binomial model structure with the number of migrating males per day as the predictor variable nested within timeframe.

To sex these animals a separate study was undertaken (supplementary results). Here, 101 biopsy samples were collected from 66 different migrating groups and the samples analysed for sex. Of the 101 samples collected, 91 (90%) were assigned a sex in the field based on their behaviour. The assigned sex was then compared to the biopsy result of sex. Of the 91 assigned a sex, only one was incorrectly assigned meaning 89% of the samples were correctly sexed based on the animal's behaviour and the group composition.

To estimate the sex ratio of migrating animals, all land-based observations of migrating groups were used. This equated to 137 observation days (from 2003 to 15) comprising of 1860 groups. Each member within each group was allocated a sex based on group composition and behaviour and the sex ratio of migrating animals estimated. A sensitivity analysis was carried out to estimate the effect of allocating animals of unknown sex male or female on the estimated number of migrating males per day. To check for any systematic bias, wthe proportion of migrating animals assumed to be male for day was compared between study years using a general linear model (with the proportion of estimated males as the response variable and year as the fixed effect and assuming a quasipoisson distribution).

Research sample

Data collection for each timeframe occurred during the annual migration of humpback whales, from breeding grounds in the Great Barrier Reef, to feeding grounds in the Antarctic Ocean. The study site was located off the coast of Peregian Beach (north of Brisbane, in Queensland, Australia), which was approximately one-third of the way along their return migration route. Migrating groups were tracked visually (7am to 5pm, weather permitting) from a land-based elevated survey point, Emu Mountain (73m elevation). For this study, a group was defined as cluster of whales within 100 m of each other that were diving and surfacing together. Groups consisted of lone singing animals, lone animals, female and calf pairs, female-calf and male escort(s), adult-only group with the number of adults. Lone adult males (singing or not singing) were targeted for the study given the study was aimed at breeding tactics in male humpback whales.

A total of 101 adult humpback whales were biopsy sampled during the 2014/2015 field effort to represent the sex ratio of migrating adults. Overall, 30 females and 71 males were sampled equating to a male dominated sex ratio of 1: 2.2 indicating a male-dominated migrating population.

Sampling strategy

Four datasets, equating to four post-whaling timeframes, were used for this study: 1997 (32 years post-whaling), 2003/2004 (38/39 years post-whaling), 2008 (43 years post-whaling and 2014/2015 (49/50 years post-whaling). Data collection for each timeframe

occurred during the annual migration of humpback whales, from breeding grounds in the Great Barrier Reef, to feeding grounds in the Antarctic Ocean. Datasets were dependent on funding availability. The sample size was reliant on the number of migrating animals therefore there was no predetermined sample size.

For the biopsy study, all available groups were approached and sampled and the sample size was a product of how many animals could be successfully biopsied and the time available for sampling (effort). As many animals as possible were sampled within the time.

Data collection

Migrating groups were tracked visually (7am to 5pm, weather permitting) from a land-based elevated survey point, Emu Mountain (73m elevation). A theodolite (Leica TM 1100) was used in conjunction with a notebook computer running Cyclopes software (E. Kniest, Univ. Newcastle, Australia) to track the groups in real-time and note group behaviours. The field of view was approximately 20 km in a north/south direction and 10 km offshore. Humpback whale groups were observed ad lib and tracked by teams of five people. When whale groups surfaced, the observers called the sighted behaviour, compass bearing, and angle from the group to the horizon (in reticules). Each observation included group identification letter, the time, group size and composition, whether a calf was present, direction of travel, and group location, either by using a binocular reticular measurement or a theodolite measurement. Joining and splitting animals were also noted. A join was defined as one of more animals actively moving towards a group to surface within 100 m and then match the group surfacing times. Examples of this include an individual singing or non-singing whale actively moving towards, and then joining, another individual or group of whales. If more animals subsequently moved in and joined the group, this was termed an additional join to that group. On rare occasions a singing whale remained in one place but was joined by another individual. This was termed an additional join given there was no evidence the singer actively moved to join this animal. However, the rarity of these occurrences meant the allocation of this behaviour to additional join, rather than join, had no influence on the results.

Some of the migrating animals were biopsied during the day for post-field later sexing. Note biopsied animals were sometimes part of different studies occurring at the field site (Mingramm et al. 2020; Smith et al. 2008;) and were not necessarily the animals used in this study. However, these biopsy results were used to test assumptions made in this study regarding the sex of joining whales and whales within the observed groups (see later and supplementary results for further details). Weather was noted hourly.

Acoustic recordings were made from three to five hydrophone buoys moored in 18 – 28 m of water and arranged in a line or T-shaped array. Each hydrophone buoy consisted of a surface buoy containing a custom-built pre-amplifier (+20 dB gain) and 41B sonobuoy VHF radio transmitter. A High Tech HTI-96-MIN hydrophone with built-in +40 dB pre-amplifier was suspended approximately 1 m above each buoy's mooring. Signals were received onshore at a base station 1.5 to 2.5 km away using a directional Yagi antenna and type 8101, four-channel sonobuoy receiver. Singing whales were located by cross-correlating the same song sound arriving at the different hydrophones to determine time-of-arrival differences. This was done by a trained observer onshore or during post-processing. These differences, together with an accurate knowledge of the positions of the hydrophones, were then used to determine the most likely location of the singer. Singers generally move slowly and calculating an acoustic position approximately every 10 min produced a detailed track of the singer.

Adult humpback whales were biopsy sampled during the 2014/2015 field effort. Groups of whales were approached by the research vessel for the purposes of biopsy sampling. Before sampling took place, behavioural observations were undertaken for at least 20 minutes. During this time, all animals within the group were allocated an individual identification name based on the shape and coloration of their dorsal fin. Photographs were taken of each adult's dorsal fin, fluke (if presented) and any other obvious markings. Using these individual identification markings, during a surfacing event, data such as the position of each animal in the group, and its general behaviour, was recorded (see Table 1). Animals usually kept the same position within the group, and displayed the same suite of behaviours, throughout the observation period, meaning these data could be summarised for all individuals within the group. During this observation period, the group composition was also described, as well as changes to this composition due to joining of new whales. Following this observation period, animals were biopsied.Biopsy sampling was conducted after the observation period using a PAXARMs remote biopsy system (PAXARMs New Zealand Ltd). Collected tissue samples were rinsed with distilled water, stored in 5 mL polystyrene tubes (LBSSP2002, ThermoFisher, Australia), placed briefly on ice (1 – 8 hours) and then transferred to -20°C at the end of a day. The first sampled animal was not a targeted group member, but the first animal to come close enough to the vessel to enable sample collection. This animal was then identified by the onboard observers from its dorsal fin and, if available, fluke as described above. From then on, animals within the group were purposely targeted based on their individual identification markings. This was to ensure the same animal was not re-sampled and as many animals as possible within the group were sampled. If there was confusion as to which group member was sampled, sampling videos were matched to group members based on their identification markings post-sampling. Once the group displayed obvious avoidance behaviours, sampling ceased. All tissue samples were then transferred into -80°C at the end of a field season (3 – 6 weeks duration). Genetic tests were conducted on all skin samples to determine the sex of each whale (Mingramm et al. 2020). These analyses were performed by the Animal Genetics Lab (UQ) and the Australian Antarctic Division.

Timing and spatial scale

Four datasets, equating to four post-whaling timeframes, were used for this study: 1997 (32 years post-whaling), 2003/2004 (38/39 years post-whaling), 2008 (43 years post-whaling and 2014/2015 (49/50 years post-whaling). Data collection for each timeframe occurred during the annual migration of humpback whales, from breeding grounds in the Great Barrier Reef, to feeding grounds in the Antarctic Ocean. The study site was located off the coast of Peregian Beach (north of Brisbane, in Queensland, Australia), which was approximately one-third of the way along their return migration route. Here, humpback whales were still exhibiting breeding behaviours such as singing, males joining females as escorts, and males forming competitive groups around a central female. Field work took place from the 14th September to 31st October of each year. Generally, the number of migrating groups increased per day to peak during late September and early October. Numbers then gradually fell until the end of the migration. As data collection was dependent on project funding, there are gaps. However the temporal period is spread evenly from 32 to 50 years post-whaling covering a population increase from approximately 3700 whales to approximately 27,000 whales.

Data exclusions

No data were excluded

Reproducibility

Observational study on a wild population of humpback whales post-whaling. No attempt was made to carry out the study on another

Reproducibility	population.
Randomization	Observational study on a wild population of humpback whales. Migrating humpback whales were allocated to group compositions based on observed group members. Lone individual males were sampled based on the fact they could be confirmed as males. Biopsy sampling targeted as many groups as possible where any group that was encountered was attempted to be sampled.
Blinding	Data were collected by teams of volunteers. They were blind to the study design, aims, hypotheses and analysis and were tasked with collecting observational data on migrating humpback whale groups.

Did the study involve field work? ☒ Yes ☐ No

Field work, collection and transport

Field conditions	Weather temperatures ranged from approximately 20 degC to 30 deg C. Observations were not carried out in rainfall nor in sea states over 4 (approximately wind speeds of 12 knots or over). Cloud ranged from clear skies to 70% cloud cover.
Location	East coast of Australia, Peregian Beach (north of Brisbane, in Queensland, Australia). 26.4778° S, 153.0949° E for land-based survey site. Study area extended to approximately 26.3961° S, 153.0894° E north and 26.5291° S, 153.0910° E south of Peregian Beach and approximately 10 km offshore.
Access & import/export	Emu Mountain (land based station) has public walking tracks therefore no permission required to access. No sampling transport permits required given all sampling occurred within Queensland waters under Queensland permits. Data collection on whales was from a land-based station. Biopsy samples were kept within Queensland state and were taken under Queensland state permits.
Disturbance	Whale data collection was from land therefore no disturbance would have occurred. For biopsy sampling, members were sampled as quickly as possible after first encountering the group. Once the group displayed obvious avoidance behaviours, sampling ceased.

Reporting for specific materials, systems and methods

We require information from authors about some types of materials, experimental systems and methods used in many studies. Here, indicate whether each material, system or method listed is relevant to your study. If you are not sure if a list item applies to your research, read the appropriate section before selecting a response.

Materials & experimental systems

n/a	Involved in the study	
☒	☐	Antibodies
☒	☐	Eukaryotic cell lines
☐	☐	Palaeontology and archaeology
☐	☒	Animals and other organisms
☒	☐	Human research participants
☒	☐	Clinical data
☒	☐	Dual use research of concern

Methods

n/a	Involved in the study	
☒	☐	ChIP-seq
☒	☐	Flow cytometry
☒	☐	MRI-based neuroimaging

Palaeontology and Archaeology

Specimen provenance	*Provide provenance information for specimens and describe permits that were obtained for the work (including the name of the issuing authority, the date of issue, and any identifying information).*
Specimen deposition	*Indicate where the specimens have been deposited to permit free access by other researchers.*
Dating methods	*If new dates are provided, describe how they were obtained (e.g. collection, storage, sample pretreatment and measurement), where they were obtained (i.e. lab name), the calibration program and the protocol for quality assurance OR state that no new dates are provided.*

☐ Tick this box to confirm that the raw and calibrated dates are available in the paper or in Supplementary Information.

Ethics oversight	*Identify the organization(s) that approved or provided guidance on the study protocol, OR state that no ethical approval or guidance was required and explain why not.*

Note that full information on the approval of the study protocol must also be provided in the manuscript.

Animals and other organisms

Policy information about studies involving animals; ARRIVE guidelines recommended for reporting animal research

Laboratory animals	The study did not involve laboratory animals.
Wild animals	Migrating groups of humpback whales consisting of a mix of adult male, adult female and calfs born in that year. No animals were

Wild animals	caught or transported. The likely sex ratio of groups observed was approximately 1: 1.5 (female: male).
Field-collected samples	The study did not involve samples collected from the field apart from biopsy samples (small samples of animal blubber). These samples were placed briefly on ice (1 – 8 hours) and then transferred to -20°C at the end of a day. Analyses were performed by the Animal Genetics Lab (UQ).
Ethics oversight	The University of Queensland Animal Ethics Unit provided ethical oversight of the project.

Note that full information on the approval of the study protocol must also be provided in the manuscript.
